# Pathogenesis of Zika Virus-Associated Embryopathy

**DOI:** 10.1089/biores.2016.0004

**Published:** 2016-06-01

**Authors:** Anthony R. Mawson

**Affiliations:** Department of Epidemiology and Biostatistics, School of Public Health (Initiative), Jackson State University, Jackson, Mississippi.

**Keywords:** embryopathy, Guillain-Barré syndrome, hypervitaminosis A, liver, microcephaly, pregnancy, retinoids, virus, Zika

## Abstract

A strong causal association has become evident between Zika virus (ZIKV) infection during pregnancy and the occurrence of fetal growth restriction, microcephaly and eye defects. Circumstantial evidence is presented in this paper in support of the hypothesis that these effects, as well as the Guillain-Barré syndrome, are due to an endogenous form of hypervitaminosis A resulting from ZIKV infection-induced damage to the liver and the spillage of stored vitamin A compounds (“retinoids”) into the maternal and fetal circulation in toxic concentrations. Retinoids are mainly stored in the liver (about 80%) and are essential for numerous biological functions. In higher concentration, retinoids are potentially cytotoxic, pro-oxidant, mutagenic and teratogenic, especially if sudden shifts occur in their bodily distribution. Although liver involvement has not been mentioned specifically in recent reports, conventional liver enzyme tests underestimate the true extent of liver dysfunction. The proposed model could be tested by comparing retinoid concentration and expression profiles in microcephalic newborns of ZIKV-infected mothers and nonmicrocephalic newborn controls, and by correlating these profiles with measures of clinical severity.

## Introduction

Zika virus (ZIKV) is one of a family of flaviviruses that includes West Nile, dengue, yellow fever, chikungunya, Japanese encephalitis and tick-borne encephalitic viruses.^1^ The virus is transmitted primarily by *Aedes aegypti* mosquitoes but the possibility of sexual transmission is also recognized.^2^ Zika was first identified in rhesus monkeys in Uganda in 1947 and has since spread widely throughout Africa, southeast Asia and the western Pacific, and most recently central and south America.^3^ There is now evidence of a strong causal association between ZIKV infection occurring during early pregnancy and microcephaly (defined as head circumference ≥2 standard deviations below the mean for sex and gestational age at birth), other abnormalities of the brain, fetal growth restriction, eye defects, and pregnancy loss.^4^ The underlying pathophysiological mechanisms are uncertain. This paper presents the hypothesis that the pathogenesis of ZIKV infection-associated embryopathy involves cholestatic liver damage in early pregnancy and the spillage of stored vitamin A compounds into the circulation in toxic concentrations.

## Investigations of Zika Infection-Associated Embryopathy

Thirty-five cases of microcephaly were described following an outbreak of ZIKV infection in northeast Brazil in early 2015. Most of the mothers of infants with microcephaly (74%) reported a rash during the first or second trimester. During their pregnancy, all had resided in or traveled to areas where ZIKV was circulating. Most of the infants had severe microcephaly, with widespread brain calcifications in the periventricular, parenchymal and thalamic areas and basal ganglia. Ventricular enlargement with cortical/subcortical atrophy was also reported, suggesting cerebral growth arrest.^5^

By January 2016, a total of 3530 infants with suspected microcephaly had been reported, many born to women who lived in or had visited areas of ZIKV transmission. Based on the peak number of reported cases of microcephaly in Brazil, and an assumed average duration of pregnancy of 38 weeks, the first trimester of pregnancy was temporally associated with reports of cases of febrile rash compatible with ZIKV disease in pregnant women.^6^

A prospective study of 88 pregnant women in whom a recent rash had developed found that 82% tested positive for ZIKV, with a descending pruritic and macropapular rash, conjunctival involvement and lymphadenopathy. Fetal ultrasound detected abnormalities in 29% of the ZIKV-positive women and none in the controls. Adverse findings included two fetal deaths, five cases of *in utero* growth restriction with or without microcephaly, five fetuses with ventricular calcification or other CNS lesions, and four with abnormal cerebral and umbilical arterial blood flow.^7^ Microcephaly in this cohort was part of an overall symptom profile of fetal growth restriction, cerebral calcification and eye problems that resembled rubella infection. These symptoms included pruritic rash, arthralgias, lymphadenopathy with low-grade fever in mothers, and severe growth restriction with microcephaly in the fetus. The typical clinical presentation of ZIKV infection has also been likened to mild dengue fever, but without hemorrhagic fever or death.^8^ The main associated clinical features of ZIKV are low-grade fever, maculopapular rash and conjunctivitis lasting 2–7 days.^9^

A causal association between ZIKV infection and microcephaly is further supported by a report on a large zika outbreak in French Polynesia. As many as 66% of the population was estimated to be infected (*n* = 270,000 in 2013). Of eight cases of microcephaly reported, seven clustered around the end of the outbreak, the period of highest risk being the first trimester of pregnancy, yielding an estimated risk of microcephaly of about 1%.^10^ However, the rate of other abnormalities and forms of brain damage could be much higher in ZIKV-associated pregnancies, possibly comparable to that of the congenital rubella syndrome, which ranged from 38% to 100% of mothers infected in the first trimester of pregnancy.^11^ ZIKV therefore represents a major public health problem because of the high rate of infection in the community. Substantial evidence thus indicates that ZIKV can be transmitted from mother to fetus during pregnancy; for example, ZIKV RNA has been identified in the amniotic fluid of mothers whose fetuses had cerebral abnormalities, as shown by ultrasonography; and both viral antigen and RNA have been identified in brain tissue and placentas of children who were born with microcephaly and died soon after birth.^1^

An expectant Brazilian mother who had a febrile illness with rash at the end of her first trimester was found at 29 weeks of gestation to have a fetus with microcephaly with brain calcifications. An autopsy performed on the aborted fetus revealed multifocal dystrophic calcifications in the cortex and subcortical white matter and mild focal inflammation. The absence of virus and of pathological changes in other organs suggested a strong neurotropism of the virus^12^; this was recognized in earlier studies of the brain of infected suckling mice, which revealed neuronal degeneration, cellular infiltration and softening in the brain, with virus replication in astroglial cells and neurons.^13,14^ Brain and eyes have also been described as major targets among the few reports of teratogenic effects of other flaviviruses.^15^

ZIKV exposure in pregnancy is also associated with severe fetal ocular findings. In a series of 29 Brazilian infants with microcephaly and a presumed diagnosis of congenital ZIKV, ocular abnormalities were present in 10 children (34.5%). Bilateral abnormalities were found in 7 of the 10 infants presenting with ocular lesions, the most common of which were focal pigment mottling of the retina, chorioretinal atrophy, and optic nerve abnormalities.^16^ Other reported ophthalmological findings have included cataract, asymmetrical eye sizes, intraocular calcifications, macular atrophy—well-defined macular neuroretinal atrophy and/or macular pigment mottling and foveal reflex loss—and lens subluxation.^17,18^

In addition to effects on the fetus, ZIKV infection has been linked to Guillain-Barré syndrome, an acute inflammatory demyelinating polyneuropathy which typically occurs after minor viral and bacterial infections. The syndrome usually begins with tingling and weakness in the feet and legs and spreads to the upper body and arms, often evolving into paralysis over a 4-week period, with accompanying disturbances of sensation and cranial nerve function. The risk of Guillain-Barré syndrome increases with age, with a predilection for males.^19^ The first report was recently published providing evidence of a causal link between ZIKV infection and Guillain-Barré syndrome.^20^

## Hypothesis

Circumstantial evidence is presented here in support of the hypothesis that the fetal manifestations of ZIKV infection result from an endogenous form of hypervitaminosis A due to infection-induced cholestatic liver damage in early pregnancy and the spillage of stored vitamin A compounds (collectively termed retinoids) into the maternal and fetal circulation in toxic concentrations. This process is postulated to result from interactions between the ZIKV genome and endogenous retinoids, leading to retinoic acid receptor (RAR)-induced activation of hepatic stellate cells and damage to the maternal liver; furthermore, liver damage and exposure of the fetus to excess retinoid concentrations in the early weeks of pregnancy is hypothesized to cause overall fetal growth arrest, microcephaly and other congenital anomalies.

Retinoids are mainly derived from the diet and are essential for multiple biological functions.^21,22^ Retinoic acid (RA), the active form of vitamin A in most cellular systems, is a fat-signaling molecule that binds to and activates the transcription of many target genes via the RARs and the retinoid X receptors.^23^ Retinoids in low concentration act as growth factors, whereas higher concentrations can be cytotoxic, pro-oxidant, mutagenic and teratogenic.^24,25^ About 80% of vitamin A is stored in the liver and can last for up to 2 years.^26^ Sudden shifts in the bodily distribution of vitamin A would therefore be expected to result in severe toxicity.

**FIG. 1.**
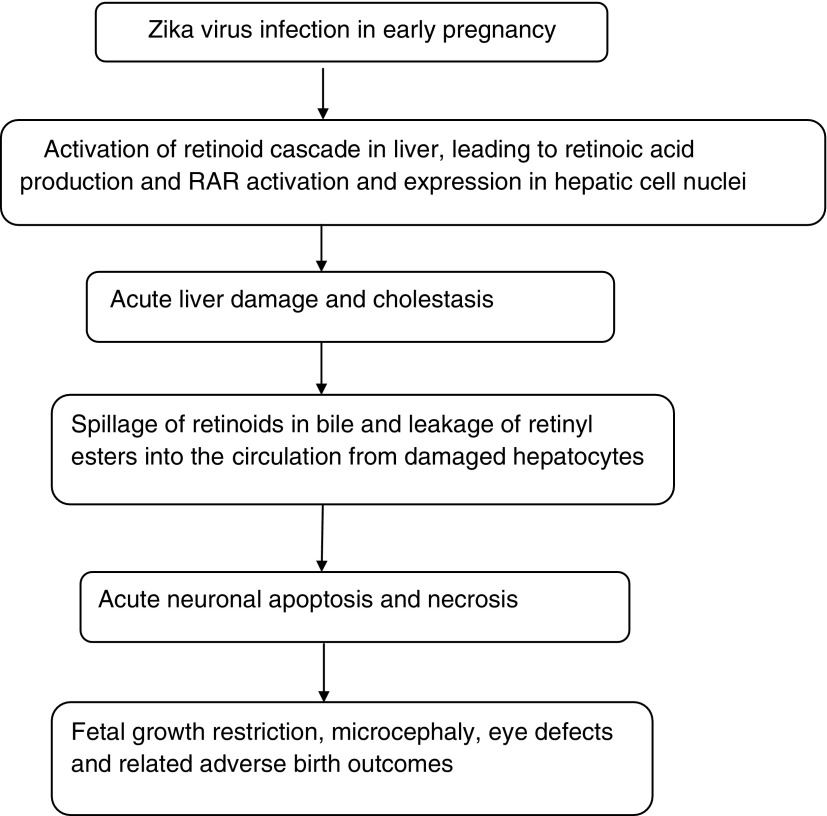
Proposed model of zika virus-associated embryopathy.

It is hypothesized that ZIKV infection induces retinoid metabolism in the liver, thereby increasing RA production and RAR activation within the hepatic cell nuclei, resulting in inflammation and liver damage. On this hypothesis, ZIKV interacts with and becomes genetically coupled to RAR receptors within the liver cell nuclei, inducing RAR activation similarly, for example, to human hepatitis B virus,^27^ HIV^28^ and human cytomegalovirus.^29^ Although liver dysfunction and abnormal liver function tests have not been specifically mentioned in recent reports, ZIKV was isolated from two out of three patients with jaundice in whom infection with malaria and yellow fever virus were ruled out during an investigation of yellow fever in Eastern Nigeria.^30^ Conventional liver enzyme tests also underestimate the true extent of liver dysfunction.^31^ (Fig. 1).

The hypothesis that ZIKV induces hepatic inflammation and tissue damage via increased RAR activation is consistent with the known role of excess vitamin A in causing liver damage.^32^ Polar retinol metabolites extracted from liver tissues of rats caused hepatocyte damage in a concentration- and time-dependent manner, due to apoptosis.^33^ Vitamin A activates Kupffer cells through INF-γ production by activated T-lymphocytes.^34^ In a mouse model of dengue hemorrhagic fever, in which one of the most affected organs is the liver, the major effects were steatosis, hepatocyte swelling, necrosis and plasma leakage, with significant increases in liver enzyme levels.^35^ In cholestasis, vitamin A metabolites spill into the circulation in bile and retinyl esters leak from damaged hepatocytes.^36^ Acute vitamin A toxicity is associated with normal or low circulating concentrations of retinol due to impaired hepatic mobilization and secretion, with increased fractions of retinyl esters circulating with plasma lipoproteins, unbound to retinol-binding protein (RBP). Symptoms usually disappear after withdrawal of vitamin A, except for occasional liver enlargement.^24^

## Similarities to Hypervitaminosis A

Strong parallels between the manifestations of ZIKV infection and those of hypervitaminosis A can be seen in the RA syndrome associated with the treatment of acute promyelocytic leukemia with RA,^37^ and in the effects of vitamin A supplements or excess dietary intakes of vitamin A-containing foods such as liver.^38^ Symptoms of acute vitamin A poisoning include fever, arthralgia and arthritis, myalgia, headache, flu-like symptoms, conjunctival congestion, lymphadenopathy, pruritus, erythematous rash, weakness, anorexia, skin peeling and altered mental status. Less common effects include hepatosplenomegaly, miscarriage, Guillain-Barré syndrome^39^ and thrombocytopenic purpura.^37^ Likewise, bone pain, fatigue^40^ and headache are major symptoms of hypervitaminosis A.^41^

Retinoids play a vital role in embryogenesis, acting as morphogens through concentration gradients in RA.^42^ RA acts on the cell nucleus to change the pattern of gene activity by binding to specific ligand-activated RARs. Retinaldehyde dehydrogenases catalyze the synthesis of RA from retinol and determine the spatial and temporal concentration gradients of RA required for normal embryonic development.^43^ If consumed or administered early in pregnancy, retinoids can also cause a wide variety of congenital defects in animals and humans, depending on the stage of gestation, dose, and route of administration,^42,44^ including fetal resorption and stillbirth. Even low intakes of vitamin A in early pregnancy (7800 μg/day) are associated with congenital malformations.^45^

Abrupt arrest of fetal growth^46^ and growth restriction in infants^47^ are known effects of RA and hypervitaminosis A. Rats that were administered 14 mg/kg of all-trans-RA for 13 weeks showed signs of growth arrest, anemia, elevated serum alkaline phosphatase, bone fracture and testicular degeneration.^48^ Microcephaly in particular is a recognized malformation of the central nervous system associated with hypervitaminosis A in early pregnancy.^49^ Benke^50^ described two infants with microcephaly, frontal bossing, hydrocephalus, microphthalmia and small, malformed and low-set undifferentiated ears whose mothers had taken the drug isotretinoin in the first trimester of pregnancy. Recalling reports of ZIKV-associated microcephaly and calcifications in the fetal brain and placenta,^12^ RA regulates calcification of mammalian limb cartilage^51^ and excess dietary intake of vitamin A promotes heart valve calcification *in vivo*.^52^

With regard to eye defects, cataract is an established feature of retinoid toxicity.^53^ Both prenatal and postnatal exposure to isotretinoin are associated with retinopathy and optic nerve abnormalities.^54^ RA contributes to light-induced retinopathy in mice via plasma membrane permeability and mitochondrial poisoning, caspase activation and apoptosis.^55^ Mutations involving a loss of retinol dehydrogenase (RDH12) have been linked to severe retinal dystrophy, involving light-induced retinal apoptosis in cone and rod photoreceptors. RDH12 shifts the retinoid balance toward increased concentrations of retinol and decreased bioactive RA, which protects against retinaldehyde-induced cell death and correlates with reduced RA concentrations in RDH12-expressing cells. RDH12 thus acts as a regulator of RA biosynthesis, protecting photoreceptors against enzymatic overproduction of RA.^56^

## Conclusions

The hypothesis proposed here is that ZIKV infection-associated fetal growth arrest, microcephaly and related congenital anomalies, as well as the Guillain-Barré syndrome, are due to mild liver damage and resulting perturbations in retinoid metabolism during the critical period of embryogenesis. The hypothesis could be tested by comparing retinoid concentration and expression profiles in microcephalic newborns of confirmed ZIKV-infected mothers and nonmicrocephalic newborns, and by correlating these profiles with measures of clinical severity.

Microcephaly and other fetal abnormalities can be detected at 18–20 weeks of gestation by ultrasonography.^7^ Subject to testing, the hypothesis suggests the possibility that increased risks of adverse pregnancy outcomes could be detected earlier in pregnancy through serum retinoid profiling. Based on such data, it may be possible to reduce the risk of ZIKV-associated adverse clinical outcomes, including fetal growth arrest and microcephaly, by lowering circulating concentrations of retinoids. For instance, plasma retinol and its transporter RBP can be reduced by phlebotomy and/or plasmapheresis.^57,58^ While the safety of these procedures in early pregnancy is not well defined, their judicious use could potentially reduce risks of adverse fetal outcomes as well as the more severe clinical features of ZIKV infection.

## Abbreviations Used

RA: retinoic acid
RAR: retinoic acid receptor
RBP: retinol-binding protein
ZIKV: zika virus
